# Civilisation and Obstetrics

**Published:** 1899-10-21

**Authors:** 


### CIVILISATION AND OBSTETRICS.

Starting with the theory that all our peculiarities are the result of heredity, of the survival of the fit, and of the rapid dying out of all who possess peculiarities which are detrimental, it is easy to arrive at the conclusion that all charity which helps the feeble to live on, and all medical science which brings back the sick to health tend directly to the deterioration of the race. In this month's *National Review* we have an extreme expression of this idea in a paper on "The Cult of Infirmity," by Mr. Arnold White, in which he rails at hospitals, at district nursing, and at all help given to the weak as officiously interfering with those natural laws by the operation of which the stamina of the English race would otherwise be kept up. In the same vein Dr. Archdall Reid, at the recent meeting of the British Medical Association, insisted that among the causes which render the labours of civilised women difficult must be reckoned the care which for so many generations has been bestowed upon women in their confinements among civilised nations. He says that savage women generally have large pelves and bear children with small heads. Hence the ease of their labours. When, however, a savage woman varies from her parent in such a manner as to have a small pelvis, or to bear a large-headed child, her life is endangered. A really difficult labour requiring surgical aid of course to her means death; but even a labour as difficult as is normal among civilised races would among nomadic savages certainly be fatal. The race is thus strictly purged of all narrow hips and large heads, and the right proportion between head and pelvis is maintained. But with the rise in civilisation greater care is bestowed on lying-in women; those who would otherwise have died are preserved, and thus the just proportion between head and pelvis is gradually lost. Natural labours become difficult, and difficult ones impossible without the aid of the obstetrician. Let the obstetrician, then, "think how many small-hipped women and large-headed children he saves annually," and of the harm he does to future generations!

